# Efficacy of irrigation tubes in the management of para rectal cavities associated with complex fistula-in-ano

**DOI:** 10.1186/s12893-018-0430-3

**Published:** 2018-11-09

**Authors:** Supul Banagala, Umesh Jayarajah, Isuru Almeida, Dharmabandhu Nandadeva Samarasekera

**Affiliations:** 1 National Hospital of Sri Lanka, Colombo, Sri Lanka; 2 Department of Surgery, Faculty of Medicine, University of Colombo, P.O. Box 271, Kynsey Road, Colombo 8, Western Province Sri Lanka

**Keywords:** Irrigation tubes, Para rectal cavities, Fistula-in-ano

## Abstract

**Background:**

Surgical management of complex perianal fistula associated with pararectal cavities can be challenging. We hypothesised that healing of the pararectal cavities prior to healing of the fistula leads to a better outcome. We aimed to assess the efficacy of irrigation catheters in the healing of pararectal cavities associated with fistula-in-ano.

**Methods:**

This study design was consistent with IDEAL stage 2a (development) and was conducted at the Professorial Surgical Unit, National Hospital of Sri Lanka, Colombo. Thirty-two patients with complex fistulae with a pararectal cavity (detected by 2D-Endoanal ultrasounography-EAUS) were included. All patients underwent examination under anaesthesia (EUA), during which insertion of an irrigation catheter to the pararectal cavity and tagging of the primary fistula tract with a drainage seton was done. Patients were advised to irrigate with antiseptic solution and were followed-up at three weekly intervals to assess cavity reduction.

**Results:**

The majority were males (96.8%, *n* = 31). The primary fistula tract in 26 patients (81.2%) was trans-sphincteric and was inter-sphincteric in 6 patients (18.7%). Mean time of cavity contraction was 34.78 (range, 21–112) days. Complete healing was seen in 87.5% (*n* = 28), with 3 patients (9.37%) being lost to follow-up and 1 patient (3.12%) having a persistent perianal fistula after 6 months of follow-up. Those who had complete healing were followed up for a median duration of 6 (range, 3–20) months and there were no recurrences.

**Conclusions:**

Irrigation in the management of pararectal cavities yielded satisfactory results. A case control trial with larger numbers and assessment of cavity size pre and post procedure by 3D-EAUS/MRI evaluation would be necessary for more objective evaluation of the efficacy of this novel intervention.

## Background

Fistula-in-ano is a frequently encountered problems in surgical practice. Perianal fistula usually have a criptoglandular aetiology and develop from perianal gland infection. Infection of anal glands is reported to occur in about 90% of the cases [1]. Sepsis then spreads in the inter-sphincteric plane and sometimes extends into other tracts as described by Parks, forming inter-sphincteric, trans-sphincteric, extra-sphincteric and supra-sphincteric fistula tracts [2]. The majority of the fistula tracts (90–95%) are simple, which are characterized by an easily identifiable fistula tract [1]. On the contrary, complex fistulae can have multiple extensions and are occasionally associated with pararectal cavities. The majority of simple fistulae can be easily delineated and intervened without worsening continence [3, 4]. However, the treatment of complex fistulae are more difficult and challenging and the complications of treatment are more frequent [5].

In addition to the primary fistula tract, there may be associated blind extensions or para rectal cavities which are extensions of the fistula tract [6]. In this study, we focused on pararectal cavities which contain necrotic material. Treatment of para rectal cavities may be difficult as debridement of necrotic material in para rectal cavities cause large perineal wounds.

Although Park’s classification is the most widely practiced method of classification, it has certain drawbacks [2]. Notably, other associated perianal conditions such as side tracts and para rectal cavities are not included in the classification though they are an important aspect to consider in the management. The American Gastroenterological Association recently proposed a more clinically oriented classification that classifies perianal fistula in to either simple or complex fistulae [7]. A simple fistula is one that is either superficial, inter-sphincteric or low trans-sphincteric with a single tract and opening. A complex fistula is one that involves a greater portion of the sphincters such as: high trans-sphincteric, extra-sphincteric or supra-sphincteric, has multiple openings, ‘horseshoes’ or associated with a pararectal cavity and/or connects to an adjacent structure, such as the vagina or bladder [7]. Thus, perianal fistulae associated with para rectal cavities are classified as complex fistulae irrespective of the nature of the associated primary tract. This is significant as simple fistulae are associated with a better outcome compared to complex tracts.

The objectives of managing a fistula in-ano are: heal the fistula, prevent recurrence and preserve sphincter function [8]. The traditional approach to a para-rectal cavities would be opening the cavity and debridement of the necrotic material. In cases of large cavities this would give rise to several problems in the postoperative period. Firstly, it would involve a large incision and also, care of a large perianal wound would be difficult. Secondly, as the fistula tract communicates with the para rectal cavity, the perianal wound may become contaminated with faecal matter impeding wound healing. Furthermore, following debridement of a large pararectal cavity, the skin wound might close before the cavity contracts thereby leading to a recurrence. Therefore, our hypothesis was that obtaining reduction of the pararectal cavity prior to dealing with the primary fistula tract would prevent the above mentioned problems. In our unit, insertion of an irrigation tube to the pararectal cavity has been practiced to achieve cavity reduction before definitive surgery for fistula-in-ano. To the authors’ knowledge, a separate irrigation tube insertion for cavity reduction has not been previously described.

In this study, we use the Innovation, Development, Exploration, Assessment, Long-term follow up (IDEAL) framework: stage 2a (development) to report our preliminary findings [9]. The IDEAL Collaboration was formed as an international group of surgeons and methodologists to promote a shift away from the traditional uncontrolled surgical research toward planned prospective studies within an established staged process [9]. Research on surgical innovation is associated with several methodological and practical challenges related to ethical concerns with respect to patient safety, patient autonomy, and distribution of health resources. However, rigorous evaluation of new surgical interventions is achievable and necessary [9]. In IDEAL stage 2a (Development), reports include a small number of patients to describe safety of procedure, short-term outcomes, indications and technical details and may describe modifications [9]. The aim of our study was to assess the efficacy of irrigation tubes in the healing of para-rectal cavities associated with fistula in-ano.

## Methods

### Study design

This is a prospective study conducted in the University Surgical Unit at the National Hospital of Sri Lanka between 2011 January and 2016 January. Ethical approval was obtained from the Ethics Review Committee of the National Hospital of Sri Lanka, prior to the study. The design was consistent with a stage 2a (development) study described in the IDEAL framework [9]. Informed written consent was obtained after explaining the study procedure, assessments, and required compliance.

### Study population

A total of 486 patients were managed for fistula in-ano between January 2011 and January 2016, of these 71 patients (14.6%) were identified to have a para rectal cavity. A total of 32 patients (6.6%) had considerably large pararectal cavities (diameter greater than 4 cm) and were deemed suitable for insertion of irrigation catheters. Other inclusion criteria were age between 18 and 75 years. Body mass index of less than 35 kg/m^2^ and willingness to participate. Exclusion criteria were vulnerable populations (eg: mentally disabled who are unable to follow instructions), those who were immunocompromised (eg: malnourished, uncontrolled diabetes mellitus, patients on long term steroids) that impair tissue healing.

### Intervention

These patients were identified as having a complex fistula with an associated para rectal cavity on Endoanal Ultrasonography (EAUS) followed by Examination Under Anaesthesia (EUA). MRI was not done due to financial constraints.

**Fig. 1:**
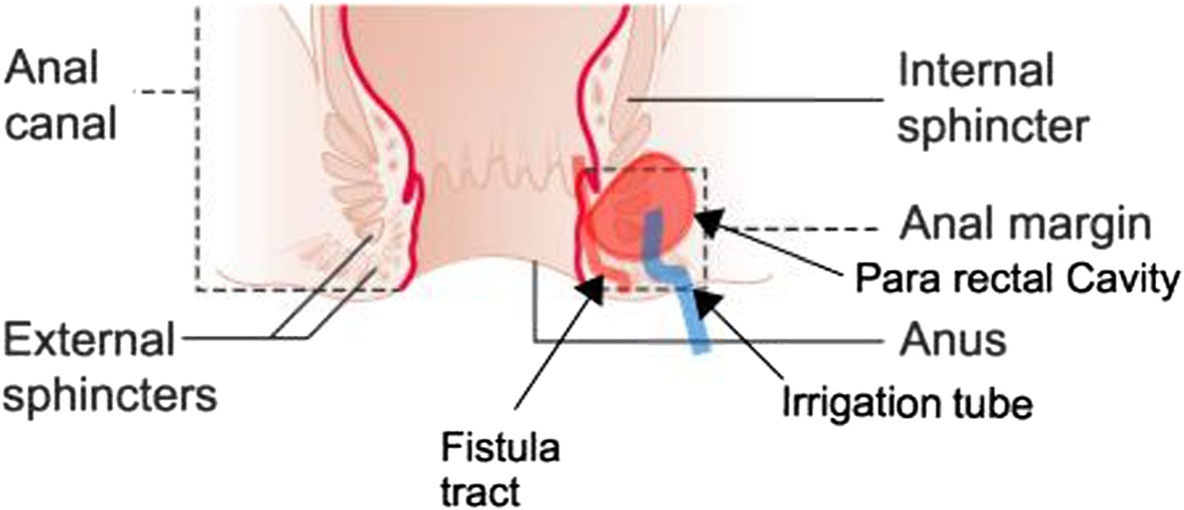
Diagram showing the position of irrigation tube in the pararectal cavity

During each EUA, gentle probing with the fistula probes and instillation of hydrogen peroxide was done to identify the internal openings and associated cavities which were already identified by EAUS. Cavities were explored through the external opening and were cleaned thoroughly of necrotic material by curettage and irrigation. Curettings from the cavities were sent for histology. During EUA, insertion of a drainage seton to the primary tract was done and insertion of an irrigation catheter was done through a separate small stab incision away from the sphincter complex. FG 6–8 feeding tubes were used as irrigation catheters and the catheters were anchored to the perianal skin with 3–0 nylon sutures (Fig. 1).

A drainage seton was inserted into the primary tract to keep it patent and no definitive procedure was done until healing or reduction of the cavity size was seen on subsequent EUAs. This protocol was followed until histology of the curettings were available (i.e to exclude TB or inflammatory bowel disease) and until significant reduction of the cavity was achieved.

Patients were advised to irrigate the cavities with an antiseptic solution, i.e. 0.25% Sodium hypochlorite solution and then povidone iodine thrice daily. Sodium hypochlorite is both an oxidizing and hydrolyzing agent that is both bactericidal and proteolytic. It has been used in irrigation of wounds and cavities from early 1900s [10]. It has also been safely used in irrigation of ischiorectal cavities following drainage of ischiorectal abscesses [11]. The 0.25% Sodium hypochlorite solution was made on request by the pharmacists. The solution can be used effectively for 6 weeks if stored in a dark bottle away from sunlight [12]. The irrigation was started when patients were inward under supervision. Patients were taught how to irrigate with correct volumes of the solution and were observed to see whether they were performing the irrigation correctly. Patients were instructed to maintain local hygiene by cleaning the perineum after irrigation three times a day. None of the patients reported difficulties in maintaining hygiene and were able to do resume day to day activities.

### Endpoints

All patients were assessed at three weekly intervals with a EUA and EAUS for a minimum period of 3 months or until complete healing of the cavity after which definitive conventional staged operations were carried out to treat the primary tract. Patients were assessed on the mean time taken for reduction of the para-rectal cavity. Contraction of the para rectal cavity was assessed by a single surgeon and significant cavity contraction was defined on the basis of EAUS and examination findings and in comparison with previous EUA measurement. Once the cavity contracted, definitive surgery was performed. All fistula tracts were laid open up to the sphincter and a cutting seton was applied to the inter/trans-sphincteric segment as the definitive surgery. Those who had complete healing of the tract were followed-up to look for recurrences.

### Procedural modification and refinement

We used a simple procedure that includes placing an irrigation tube to the associated pararectal cavity through a stab incision in the perianal skin while avoiding the sphincter complex. We did not experience any technical modification as it was a simple intervention.

### Learning curve assessment

All procedures were performed by a single senior colorectal surgeon. As the procedure was already established in our unit before the study we could not do a formal learning curve assessment. However, we believe that there is no significant learning curve as it is a simple procedure which included a perineal incision and insertion of an irrigation tube in to the cavity.

## Results

The majority of patients were males (96.8%, *n* = 31). The median age was 34 years (range: 19–68). The primary fistula tract associated with the pararectal cavity was trans-sphincteric in 81.2% (*n* = 26) and inter-sphincteric in 18.8% (*n* = 6). None of the patients had a high interpshincteric, transphincteric or suprasphincteric fistulae. All pararectal cavities were located in the ischioanal space. The minimum diameter of the pararectal cavity was 4 cm. The detailed assessment of dimensions of EAUS was not done as most pararectal cavities were irregular and was difficult to assess with 2D EAUS. The majority of the internal opening were found at or below the dentate line (96.8%, *n* = 31). An underlying aetiology was seen in 2 patients, which were Crohn’s disease and TB (Table 1).

**Table 1:** Characteristics of fistula-in-ano

Characteristic	Number
Number of patients	32
Male	31 (96.8%)
Female	1 (3.1%)
Primary fistula tract	
Trans-sphincteric with associated cavity	26 (81.2%)
Inter-sphincteric with associated cavity	6 (18.7%)
Level of fistula opening	
Below dentate line	11 (34.7%)
At the dentate line	20 (62.5%)
Above the dentate line	1 (3.1%)
Histology	
Unremarkable	30 (93.7%)
Crohn’s disease	1 (3.1%)
Tuberculosis	1 (3.1%)
Mean time of significant cavity contraction	34.78 days
Median time of significant cavity contraction	21 days (21–112 days)
Number of patients requiring reinsertion of irrigation tube (after first EUA)	8 (25%)
Outcome	
Fistula healed	28 (87.5%)
Lost to follow up	3 (9.4%)
Fistula not healed	1 (3.1%)

The majority of patients (*n* = 17, 53.1%) demonstrated significant cavity contraction at their first follow up EUA at 3 weeks. The mean time of significant cavity contraction among all patients was 34.78 (range, 21–112) days.

Three patients required reinsertion of an irrigation tube due to dislodgement. In addition, eight patients (25%) were found to have persisting para rectal cavities that were deemed large enough to require re-insertion of a new irrigation tube. The median number of definite surgical procedures (which includes fistulotomy, tagging the proximal segment with a cutting seton and subsequent retightening) was 3 (range, 1–6).

Following the contraction of the pararectal cavities and the staged operations, complete healing was seen in 87.5% (*n* = 28), with 3 patients (9.37%) being lost to follow-up and 1 patient (3.12%) having a persistent perianal fistula after 6 months of follow-up. The patient having a persistent fistula underwent a re-evaluation with EUS and EUA and underwent fistulotomy and insertion of a cutting seton. Those who had complete healing (of both pararectal cavity and fistula tract) were followed up for a median duration of 6 (range: 3–20) months and during the follow up period none had recurrences.

### Feasibility and safety

Patients complained of minor discomfort during the first week. Thereafter, they did not need analgesics and were pain free. Three patients required reinsertion of an irrigation tube due to dislodgement. There were no other significant complications related to the procedure.

## Discussion

The practice of our unit was to irrigate the pararectal cavity with 10 ml of 0.25% Sodium Hypochlorite solution thrice daily until the necrotic material had been washed out and then to convert to irrigation with 10 ml of 10% Povidone Iodine solution in a similar frequency. Though irrigation tubes have been used in the past to irrigate the fistula tracts without cavities, to our knowledge, we have not come across any study using an irrigation tube directly inserted into a cavity associated with fistula tracts [13].

It has been proven that Sodium Hypochlorite solution has a role in reducing the bioburden of wounds [14]. Thus, it is a suitable antiseptic to be used in the initial stage of irrigation to reduce the bacterial load of a para rectal cavity. Povidone iodine is useful for cleansing and prevention of infection, thus it is useful once the cavity has been cleared of necrotic material and faecal matter [14].

Patients were shown how to irrigate the cavity using a disposable syringe during their ward stay. They were also advised to return to the ward if there was any blockage or dislodgement of the tube. This is a relatively simple, low cost procedure that is acceptable to patients when compared to large perineal incisions traditionally used for debridement of necrotic material.

All EUA’s were performed by a single Consultant Colorectal Surgeon, eliminating operator dependent bias that could have occurred with multiple surgeons. One drawback of the study was that ours was a single arm study with no comparison group. The majority of patients selected were males but this was unintentional as perianal fistula shows a higher prevalence among men [15].

Important factors in the management of para-rectal cavities using this technique were, identification of the primary fistula tract, identification of side tracts or para rectal cavities, consideration of the anal sphincter complex in making perineal incisions and regular irrigation with appropriate antiseptics. No significant additional complications were noted with the procedure except for, dislodging of the irrigation tube.

In our cohort, 53.1% of patients demonstrated significant cavity contraction at their first follow up EUA at 3 weeks and the mean time of significant cavity contraction was 34.78 days. Reinsertion of the irrigation tube was required in 25% who had persisting pararectal cavities. Complete healing of fistula was seen in 87.5% (*n* = 28) and 1 patient (3.12%) had a persistent perianal fistula after 6 months of follow-up. Choi et al. [16] did a retrospective analysis of 12 patients who were diagnosed of a deep horseshoe fistula who were treated with seton irrigation and compared with those treated with conventional setons. The mean time to removal of the seton was 22 (18–29) days for self-irrigation versus 33 (28–39) days for conventional treatment (*p* < 0.001). Thus, seton irrigation was shown to shorten the period of treatment. However, the use of a separate irrigation catheter for pararectal cavities was not previously assessed. Therefore, we are unable to compare our findings with other similar studies.

### Limitations

We report the outcomes of a small cohort of patients with a short follow up and did not involve a control group. Our primary objective was to report the preliminary outcome of this procedure. Therefore, the benefits of this intervention over the conventional treatment could not be objectively assessed. A drawback of our study was the inability to measure the size of the para-rectal cavities accurately. Even the EAUS would only give a 2D measurement unless a 3D probe is used. Therefore a perineal MRI scan would provide an objective assessment of the outcomes of this study which we were unable to do due to financial constraints. Analysis of the scores regarding postoperative anal incontinence was not done which is another limitation of this study. Although we included patients from 2011 January, the senior author has been practicing this technique with good outcomes before the study period and was an established technique in our unit. However, we prospectively followed up the patients only after 2011 January to objectively measure their outcomes. Furthermore, as the study was conducted after the procedure was established, we did not assess the learning curve which is another limitation in this study. The follow-up time of 6 months is a limitation of the study and ideal follow up period may be 12 months as fistulae may recur after a period of 6 months.

### Conclusion

Though being a descriptive study, this demonstrates that irrigation tube insertion yields satisfactory results in the management of para rectal cavities.

A case control study with larger numbers and assessment of cavity size pre and post procedure by 3D endoanal ultrasound/MRI evaluation would be necessary for an objective evaluation of the efficacy of this intervention. However, we believe that this preliminary study is useful in the assessment of this novel technique in the management of complex perianal fistulae associated with pararectal cavities.

## Abbreviations

2D: Two-dimensional
3D: Three-dimensional
EAUS: Endoanal Ultrasonography
EUA: Examination Under Anaesthesia
FG: French Gauge
IDEAL: Innovation, Development, Exploration, Assessment, Long-term follow up
MRI: Magnetic Resonance Imaging
TB: Tuberculosis
